# Near-Misses and Stop Buttons in Slot Machine Play: An Investigation of How They Affect Players, and May Foster Erroneous Cognitions

**DOI:** 10.1007/s10899-017-9699-x

**Published:** 2017-07-12

**Authors:** Mike J. Dixon, Chanel J. Larche, Madison Stange, Candice Graydon, Jonathan A. Fugelsang

**Affiliations:** University of Waterloo, 200 University Ave. West, Waterloo, ON N2L 3G1 Canada

**Keywords:** Gambling, Near-misses, Stop button, Erroneous cognitions, Cognitive biases, Structural game features, Frustration, Illusion of control

## Abstract

In modern casinos, multiline slot machines are becoming increasingly popular compared to traditional, three-reel slot machines. A paucity of research has examined how the unique presentation of near-misses and the use of a stop button in multiline slot machines impact erroneous cognitions related to the perception of skill and agency during play. Our goal therefore was to determine the prevalence of erroneous cognitions pertaining to near-miss outcomes and the usage of a stop button and then to see whether the stop button affected players’ experiences of winning, losing and near-miss outcomes. We recruited 132 gamblers from a casino in Ontario. They played two versions of a slot machine simulator: one with a stop button and one without a stop button. We measured player’s arousal [skin conductance responses (SCRs), pressure on the spin-button), and behavioural responses (post-reinforcement pauses (PRPs)] to wins, losses and near-misses during play. We predicted more robust physiological SCRs and longer PRPs to wins in the stop button game. We also predicted that near-misses encountered in the stop button game would trigger greater levels of arousal and frustration in players, as indexed by larger SCRs, and greater force applied to the spin button to initiate the next spin. Erroneous cognitions pertaining to the stop button and near-misses respectively were assessed following play. Results showed that a small but meaningful percentage of players held erroneous cognitions about the stop button (13.6%) and near-misses (16%). Players depressed the spin button harder, and had larger SCRs for all outcomes when using the stop button. Players also paused longer for near-misses in the game involving the stop button. Our findings converge to suggest that the stop button encourages an erroneous perception of skill in some players, and consequentially impacts how such players perceive their outcomes in multiline slot machines.

## Introduction

Slot machine gambling has been prevalent in Canada since its legalization in 1985 (Campbell et al. 2005). Since then, slot machine games in Canadian casinos have evolved to possess an impressive array of design types and different features to entertain players. Indeed, such continuous evolvement partially explains why they have been able to maintain player interest across the decades. Despite their potential for entertainment, slot machines have been cited as being the most problematic form of gambling (Williams and Wood 2007). In fact, they typically generate more than 24% of casino revenue (Williams and Volberg 2013), and their increasing availability is positively linked to the prevalence of problem gambling in Canada, currently estimated at approximately 3% of the population (Williams et al. 2012). In 2010, a problem gambling helpline in Ontario received approximately 2000 calls. The vast majority of these calls involved concerns about slot machine gambling (Centre for Addiction and Mental Health 2011). In one particular study, 80% of high-risk problem gamblers cited slot machines as being their primary game of choice (Ladouceur et al. 2002). Given this link to problem gambling behaviour, it is imperative to explore how such problems may manifest at the origin of play.

Cognitive biases have been shown to influence the onset of problem gambling behaviour. Of important consideration are cognitive biases that distort players’ beliefs about the role of skill over game outcomes that are in actuality purely chance-dependent. One prominent subset of erroneous cognitions related to personal skill is the “illusion of control”, which is characterized by a player’s inflated sense of ability to influence their probability of winning in such games of chance (Langer 1975). Such skill-oriented cognitions are primarily defined by an internal locus of control, such that players psychologically attribute outcomes of an event to their personal control or their own abilities. Such thinking styles ultimately lead to disadvantageous decision making in gambling, as evidenced by increased bet sizes, longer gambling session lengths, and in some cases, chasing behaviour (Walker 1992; Dixon 2000; Chóliz 2010; Lim et al. 2014). Although it is known that problem gamblers are the most vulnerable to adopting skill-oriented erroneous cognitions both during gambling, and outside of the gambling context (Orgaz et al. 2013), little is known about how these erroneous cognitions may relate to specific slot machine features.

One particular slot machine feature is the so called near-miss. In a simple three-reel slots game a classic near-miss would involve two jackpot symbols on the payline, with the third just off the payline. In slots games where outcomes are determined by a random number generator, objectively no losing outcome is any “closer” to a win than any other outcome. Many players fail to appreciate this and will interpret a near-miss as being closer to a win than a regular loss (Dixon and Schreiber 2004). Others appear to interpret near-misses as indicators of skill at slots or as a harbinger of an upcoming win. One goal of this study was to assess how common such erroneous cognitions are among frequent gamblers, and whether such beliefs about near-misses are related to problem gambling.

It has been suggested that certain erroneous cognitions in chance-based games surface as a function of game features that foster active interaction by the player (Langer and Roth 1975; Wagenaar 1988). One such feature is the “stop button” mechanism, or in gambling parlance the “skill stop” device (Rothstein 2012). Normally, depressing the spin button on a slot machine causes the animated reels to spin in unison for a brief duration, then each reel will slow and eventually come to a stop with the leftmost reel stopping first followed in sequence by the remaining reels. By pressing the stop button while the reels are spinning, however, the animated reels will come to a stop far more quickly than in games without a stop button. Importantly the stop button does not permit players to manipulate *where* the reels will stop. In fact, the stopping positions of the reels are actually determined by a random number generator as soon as the player presses the spin button. Use of the stop button merely causes the reels to settle into their already-selected positions more quickly (Harrigan and Dixon 2009). What makes the stop button potentially problematic is that it may not be apparent to players that these buttons only impact the speed of play. Some players appear to think that their interactions with the stop button have an impact on where the reels stop and hence the outcomes of the spin. To our knowledge, only one study has directly tested the impact of the stop button on player’s erroneous beliefs surrounding the function of the stop button itself. Ladouceur and Sévigny (2005) examined how the stop button contributes to the illusion of control while gambling. Players played two sessions on a video lottery simulator: one that included the use of the stop button, and the other session without the stop button. A majority of players endorsed the belief that the stop button influenced where the reels stopped, with some even expressing the belief that there was actually a skill to using the stop button. Presumably such skill involved the erroneous perception that by pressing the stop button at just the right time the reels could be made to stop over winning outcomes. Crucially, in this study players chose to play twice as many spins when they had access to the stopping device compared to the game without a stop button (Ladouceur and Sévigny 2005). This suggests there may be a problematic relationship between the presence of the stop button, erroneous cognitions and play duration.

In Ontario, steps have been taken to dispel erroneous cognitions involving stop buttons. All slot machines with stop buttons have signage on the machine that explicitly tells players that stop buttons only affect the speed of play, and do not influence the game outcomes. One goal of this study was to ascertain if at least some players recruited from these casinos (who should be familiar with this message) would nonetheless still hold erroneous cognitions about the stop button.

Although the stop button does not allow players to manipulate the stopping position of the reels, several lines of research suggest that it may grant players a sense of personal agency during play. Structural features that instill a sense of agency may perpetuate the irrational belief that players’ skillful “performance” during gameplay will influence their outcomes (Witts et al. 2015). Clark et al. (2012) conducted a study using a simplified slots-like game involving two-reels each with six symbols. The game began with a symbol from reel 1 being selected as “the play icon” (e.g., a banana icon). The second reel contained this same icon along with five other different icons. The second reel then “spun” and stopped. If the second reel stopped so that the matching banana icon on reel two was horizontally adjacent to its twin on reel one the player won. If the second reel stopped so that a different symbol appeared next to the play icon (e.g., an orange, or a strawberry stopped beside the banana on reel one) they lost. There were three types of key outcomes: wins (adjacent matching icons), regular losses where the required matching symbol on reel two stopped in a position relatively far away from the winning alignment, and a near-miss where the matching symbol stopped close to—but just off the winning alignment. Typically, because the near-miss conveys a thwarted goal, they are notorious for being physiologically arousing and frustrating (Reid 1986; Brown 1986). In this two-reel slot machine task, personal agency was manipulated by either having the computer select the play icon, or alternatively having the *player* select this to-be-matched icon. When players encountered a near-miss, regardless of condition, they experienced negative affect. However, those in the personal agency condition experienced significantly more negative affect than when the computer selected the play icon (Clark et al. 2012). This amplified frustration response to near-misses was attributed to the fact that players felt like they were the “cause” of their outcomes. That is, near-misses under the condition of agency were perceived as a form of feedback confirming the adequacy of game icon choices (Clark et al. 2012). Of equal importance, even though near-misses were more aversive in the personal agency condition, they nonetheless triggered the greatest urge to continue playing game.

### The Current Study

In the current study we sought to assess whether the stop button would be capable of imbuing players with a sense of agency during play. If players do indeed misinterpret the stop button as a skill-device allowing them to having some influence over outcomes then it should, as in the Clark and colleagues study, make near-misses more frustrating. We could then replicate the important sense of agency shown by Clark and colleagues, but be able to attribute this agency to a feature that is actually available on most modern slot machines.

Rather than interrupting players during play to poll their frustration levels, we chose to use measures that could capture their emotional state without interrupting play. Moment-to-moment changes in arousal due to hedonic pleasure and frustration can reliably be quantified by changes in skin conductance responses (SCRs) (e.g., Lobbestael et al. 2008). Slot machine research has consistently shown that near-misses trigger more robust SCRs following their delivery than regular losses (Clark et al. 2012). In addition, we have shown that near-misses trigger even larger SCRs than small wins (Dixon et al. 2011) and that it takes a relatively large win to generate an SCR as large as that generated by the frustration of the near-miss (Dixon et al. 2013).

Another useful means of gauging players’ reactions to various slot machine outcomes is the post reinforcement pause (PRP). In slots play, PRPs are operationally defined as the time between the onset of an outcome’s delivery and the initiation of the next spin (Dixon and Schreiber 2004; Dixon et al. 2013; Belisle and Dixon 2016). When players spin and lose in a continuous game like slots, players tend to immediately initiate the next spin (Dixon et al. 2013). Conversely, winning in slots play is characterized by significantly longer PRPs as players stop to mentally celebrate reward attainment (Dixon et al. 2013). The relation between PRPs and near-misses is somewhat controversial. Two studies using small student samples showed that PRPs for near-misses were significantly longer than the PRPs for regular losses and more akin to the PRPs for wins (Dixon and Schreiber 2004; Belisle and Dixon 2016). By contrast, two studies that included larger community samples of high frequency slots players (including problem gamblers) showed that although near-misses generated far larger SCRs than regular losses the PRPs were either equivalent to regular losses (Dixon et al. 2015), or were slightly smaller than regular losses (Dixon et al. 2013).

A relatively novel means of measuring arousal due to excitement and frustration involves measuring the force applied to the spin button following various outcomes. In a study of 56 regular gamblers recruited from a casino conducted by Dixon et al. (2015), players pressed significantly harder on the spin button following near-misses to initiate the next spin, than the force used to trigger spins following regular losses. We assumed that such forceful spin initiations were attributable to the frustration of the near-miss. In this same study, an unexpected finding was that players also applied large forces following large wins—a finding we retrospectively interpreted as an indication of increased arousal due to the excitement caused by the big win (Dixon et al. 2015). Although we have used force to measure arousal before in this one relatively small scale study, we hoped to replicate the effects of near-misses and salient wins on force responses in a study with a larger sample size.

**Fig. 1:**
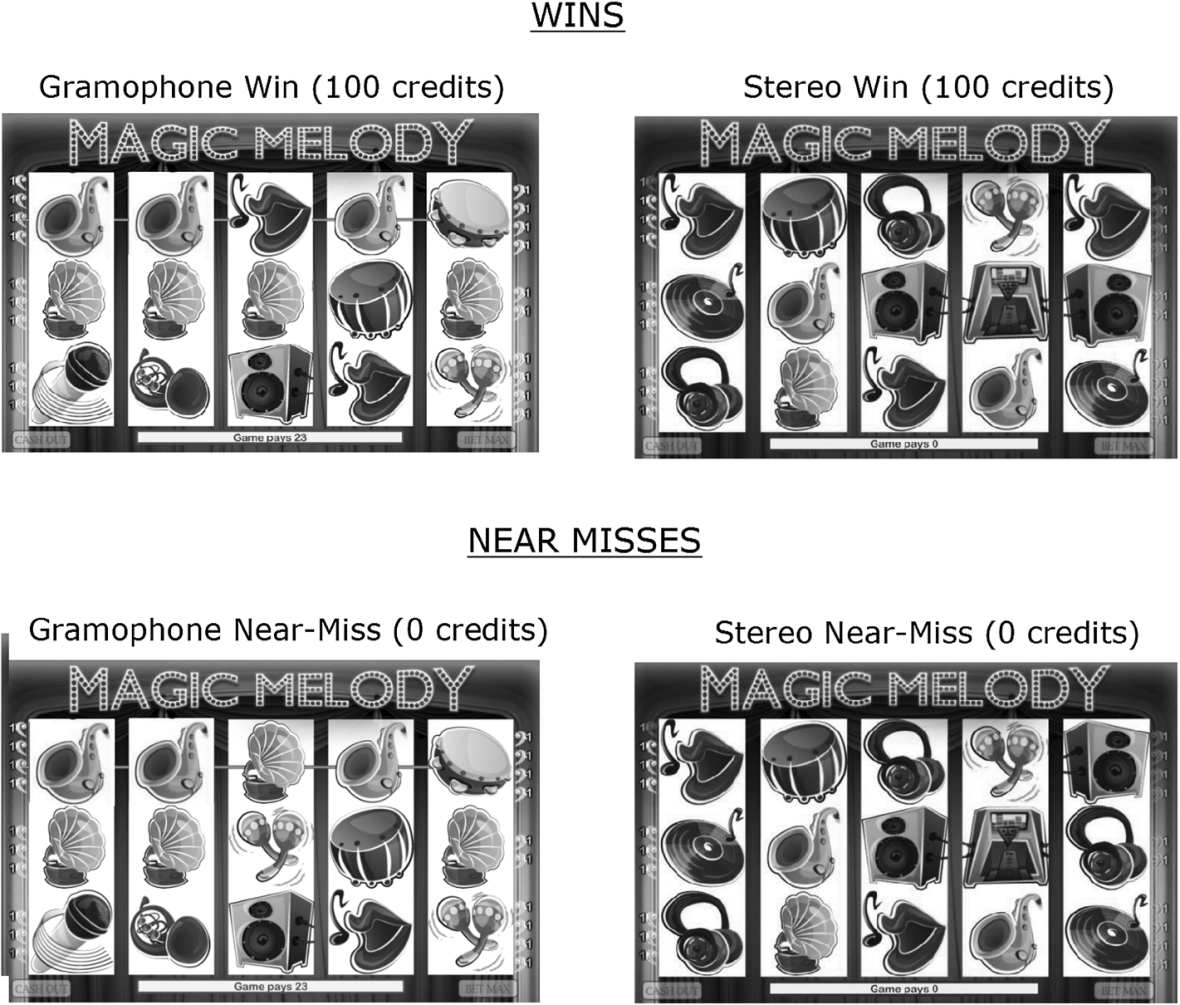
Winning and near-miss outcomes for gramophone and stereo wins (from Dixon et al. 2015)

Most research on slot machine near-misses has involved games where players hope for multiple matching symbols to fall onto a single horizontal payline. In modern casinos, however, the most heavily used slot machines are multiline slot machines where players wager on multiple lines at once. Many of these lines involve zig-zag combinations. Their complexity at first blush would seem to preclude players even noticing near-misses. Recently, however, research has shown that near-misses can impact players in these multiline games (Dixon et al. 2015; Sharman et al. 2015). In the Dixon et al. (2015) study (and in the current study), the slot machine simulator was patterned after a commercially available, five reel, multiline game. In this game, there was a unique type of large win involving a symbol group we call a “horizontal triplet”. Horizontal triplets are unusual in that these special symbols only appear on the third, fourth and fifth reels. When horizontally aligned, they form a familiar three-part object that signifies a relatively large win. The top left hand panel of Fig. 1 shows a win involving a standard symbol (three aligned gramophone symbols which in our simulator led to a win of 100 credits). The top right panel shows an example of a 100-credit horizontal triplet win. In our simulator (and in the game it was patterned after) the low-level visual features in the horizontal triplet (e.g. the straight edges, and the “connecting” wires of the stereo) caused the components of the triplet to stand out from other game symbols. In addition, the appearance of each part of the triplet (the left-speaker, the amplifier and the right speaker) was accompanied by a distinct auditory signal designed to draw players’ attention to the triplet’s components. The combination of such low-level visual features and auditory signalling made these horizontal triplets quite salient, causing wins involving the horizontal triplet to generate significantly larger SCRs and longer PRPs than standard-game wins (e.g., the gramophone) that were equated in win size. Crucially, horizontal triplets were capable of triggering near-miss effects. An example of a near-miss in the horizontal triplet is shown in the lower right panel of Fig. 1. In this study players reacted to these horizontal triplet near-misses with larger SCRs than regular losses, and applied more force to initiate the next spin than regular losses. Thus, another specific goal of this study was to replicate these effects using a larger sample of gamblers.

A final aim of the present study was to investigate erroneous cognitions pertaining to the use of the stop button, and how its use affects players’ experience of winning, losing and near-miss outcomes. First we sought to replicate the important results of Ladouceur and Sévigny (2005) which showed that at least some players held erroneous cognitions specifically related to the stop button. By combining aspects of Ladouceur and Sévigny (2005), Clark et al. (2012) and Dixon et al. (2015), we aimed to paint a contemporary picture of the influence of slot machine features on players’ erroneous cognitions and players’ physiological responses to outcomes. Importantly, since the vast majority of players prefer multiline over single line games (Livingstone and Woolley 2007; Templeton et al. 2015), we tested these hypotheses using a simulator designed to emulate a popular multiline game. We had players play two sessions on our simulator: one with a stop button feature, and the second without a stop button feature. If the use of the stop button evokes a sense of personal agency in players, then we would expect more robust physiological SCRs and longer PRPs to wins in the stop button game. Similarly, we would expect that near-misses in games where a stop button is employed would trigger greater levels of arousal and frustration in players, as indexed by greater levels of SCRs, and greater force to initiate the next spin.

## Methods

### Participants

A total of 132 participants (76 males, 36 females) were recruited from a local casino in the city of Brantford, Ontario. Participants were first asked to complete a pre-screen survey to ensure that participants: (1) Were over the age of 19, (2) had gambled on a slot machine at least once in the last 12 months, and (3) were not in treatment for problem gambling. Participants failing to meet these criteria were excluded from participating. Participants included experienced slot machine gamblers between the age of 19–86. Average age of participants was 55.88 years. Participants were excluded if there was any technical difficulty with the physiological data, survey data or if participants withdrew from the study.

### Apparatus

**Fig. 2:**
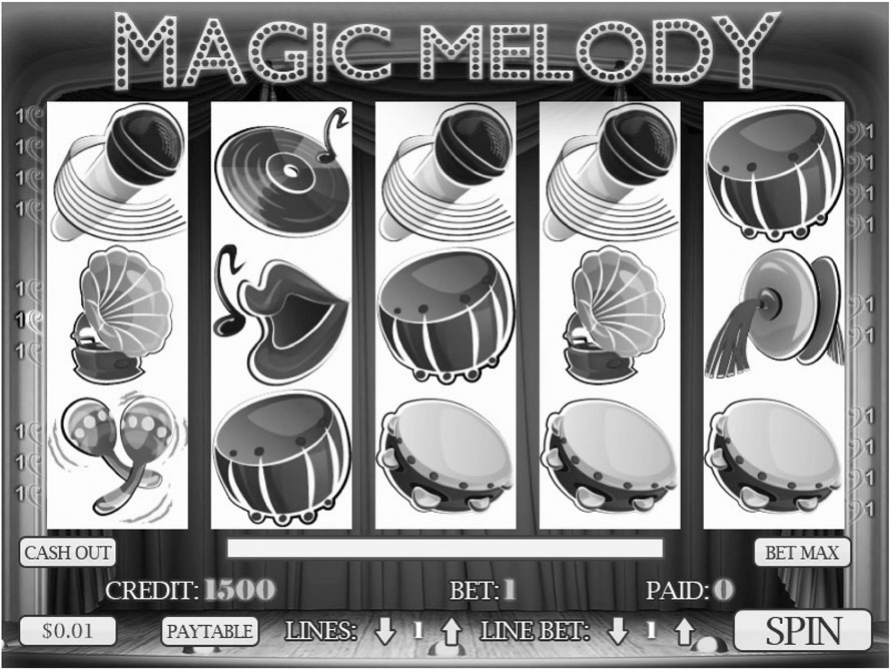
A depiction of the five-reel multiline simulator used in the current study patterned after a commercially available slot machine

**Fig. 3:**
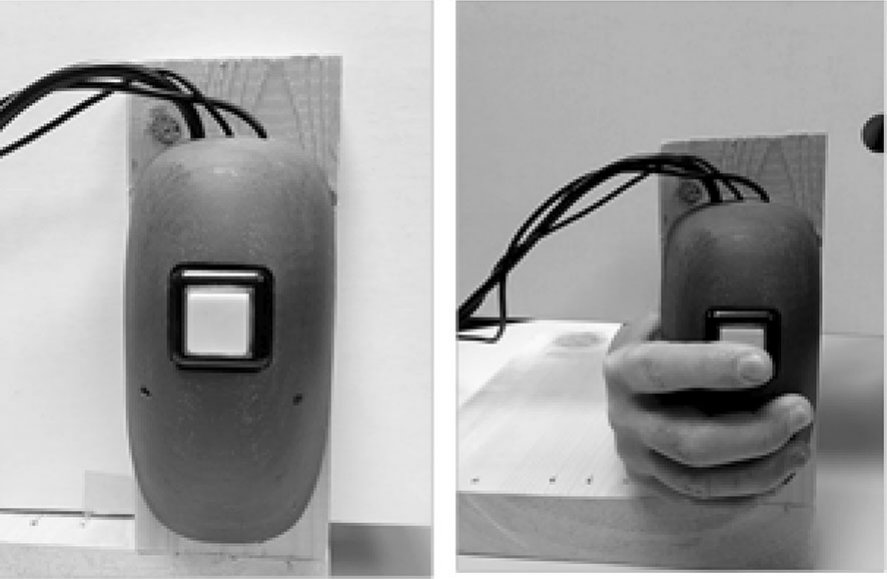
A modified mouse was placed facing away from the player so they could feel the button, but not see it. Spins were initiated by pulling on the mouse button. The stop button feature was invoked by re-pulling on the mouse button while the animated reels were spinning

*Slot Machine Simulator* Participants played a five-reel multiline slot machine simulator modelled after a commercially available multiline slot machine game. The simulator was a music themed slots game called “Magic Melody”. Like the commercially available game on which it was patterned, losing outcomes (where the player lost all of their wager) were followed by a complete absence of feedback (no animations, no sounds). Winning outcomes (where the slot machine paid out more than the wager) were accompanied by animations highlighting the winning line(s), and auditory feedback in the form of winning jingles, with the length of the auditory feedback proportional to the win size. The simulator also displayed “Losses Disguised as Wins” or LDWs (Dixon et al. 2010). On these outcomes, the player “won” credits, but the total number of credits were less than the spin wager. Even though these were net losses to the player, the simulator, like commercially available slots, accompanied these losing outcomes with celebratory feedback. The frequency of these outcomes (27%) were comparable to the rates for these outcomes in the commercial game. Sessions were played in blocks of 250 spins comprised of 35 wins, 67 LDWs and 148 losses. Among the 35 wins, there were 6 wins involving the highly salient horizontal triplet shown in Fig. 1. For comparison, there were 6 wins involving the gramophone symbol also shown in Fig. 1. This comparison stimulus was thought to be less salient since its features caused it to blend into the array rather than pop-out of the array like the horizontal triplet (Dixon et al. 2015). The horizontal triplet wins and the gramophone wins were each worth 100 credits. Losses included 88 regular losses, 30 near-misses involving the gramophone, and 30 near-misses involving the stereo horizontal triplet. The simulator displayed counters for the following: The number of credits ‘Paid’ on winning outcomes, the number of lines played, and amount bet. These counters are shown in Fig. 2. As can be seen in this figure, when the machine is waiting for the player to initiate a spin, a “spin” indicator is illuminated. Players activated spins using a modified mouse equipped with a force transducer shown in Fig. 3. To initiate a spin, players simply pulled on the button. For the stop button condition only, the display of the SPIN indicator would change to ‘STOP’ during the reel-spins. Players could activate the stop button by pulling on the external button while the reels were in motion. This would cause the reels to stop on the predetermined outcome more quickly than had the stop button not been pulled. Once the outcome was delivered, the SPIN illuminator would return to displaying the word ‘SPIN’.

### Measures

*Post-reinforcement Pauses* The delay between the delivery of an outcome (the last reel stopping) and the player’s initiation of the next spin, measured in milliseconds (ms) constituted the PRP for that outcome. The simulator was configured to send event markers to an ADInstruments Powerlab for each spin initiation (when the player would press the spin button), stop button deployment, and outcome delivery (e.g. stereo near-misses, wins, etc.).

*Force* A modified button (See Fig. 3) contained a force transducer which is sensitive to how hard players pulled the button toward them. Output from the force transducer was translated to a millivolt signal recorded by the Powerlab.

*Skin Conductance Response* Skin conductance responses (SCRs) were recorded using two metallic electrodes attached to the index and ring finger on the participants’ left hand. Participants were instructed to limit movement of their left hand while playing the slot machine simulator in order to minimize contamination of the SCR data. The electrodes fed into the Powerlab which amplified the participants’ skin conductance signal. SCRs for each outcome were calculated by defining a 3 s window beginning 1 s after the last reel stopped moving (i.e., when an outcome was delivered). SCRs for each outcome were calculated by taking the peak skin conductance within this window and subtracting the skin conductance level value at the beginning of this window. As per the recommendations of Dawson et al. (2000) a square root transformation was applied to these SCRs prior to analysis. For PRPs, force and SCRs the first and last trial in each 250-spin block (both regular losses) were not analyzed.

### Materials

*Pre-experiment Questionnaire* Participants first completed a questionnaire asking about demographic information (e.g. age, gender) as well as their gambling habits (e.g. gambling frequency in the last 12 months).

*Problem Gambling Severity Index* The problem gambling status index (PGSI; Ferris and Wynne 2001) was used to assess gambling-specific demographic information (e.g. problem gambling behaviour, negative consequences, etc.). Each of nine items of the PGSI were answered using a 4-point scale (0 = *never*, 1 = *sometimes*, 2 = *most of the time*, 3 = *always or almost always*). PGSI scores were derived by summing the responses on the nine items. Participants were stratified using the following mappings: 0 as non-problem gamblers, 1–4 as low-risk, 5–7 as moderate risk and 8 or more as problem gamblers.

*General Gambling Related Erroneous Cognitions* The Gambling Related Cognitions Scale (GRCS; Raylu and Oei 2004) was used to measure players’ erroneous or distorted cognitions, and gambling beliefs in general. The construct is composed of 23 items assessing interpretative control/biases (e.g., ‘Relating my winnings to my skill and ability makes me continue gambling’), illusion of control (e.g. ‘I have specific rituals and behaviours that increase my chances of winning’), perceived inability to stop gambling (e.g. ‘My desire to gamble is so overpowering’), gambling-related expectancies (e.g. ‘Having a gamble helps reduce tension and stress’), and predictive control (e.g. ‘Losses when gambling, are bound to be followed by a series of wins’). Participants rated how much they agreed with each statement on a 7-point Likert scale, with 1 representing *Strongly Disagree*, and 7 representing *Strongly Agree*.

*Near-miss Specific Erroneous Cognitions* In the post-game phase of the study, participants responded to two items designed to poll erroneous cognitions related to near-misses: “Near-misses reflect my skill at this slots game and indicate that I was close to winning” and “Near-misses indicate that a win is imminent”. Ratings for these statements ranged from 0 to 4 (0 =* strongly disagree*, 1 = *disagree*, 2 = *neither agree or disagree*, 3 = *agree*, 4 = *strongly agree*).

*Stop Button Specific Erroneous Cognitions* Participants rated an item designed to poll erroneous cognitions related to the stop button: “Using the stop button made wins more likely”. Players answered the item using one of the following options (0 = *strongly disagree*, 1 = *disagree*, 2 =* neither agree or disagree*, 3 = *agree*, 4 = *strongly agree*). Participants also responded to four items patterned after Ladouceur and Sévigny (2005): (1) “Do you believe that a player can influence the symbols on the screen after having activated the spin button?”, (2) “Is there a method for controlling the outcome of the game after the spin button has been activated”, (3) “Are there any strategies that could enable you to increase your chance of winning after the spin button has been activated?” 4) “If you were to obtain a winning combination would it be due to chance, skill or a combination of the two?” To facilitate the analyses, a composite score was calculated based on the number of YES responses to questions 1–3. If they endorsed “chance” to the fourth item, a zero was added to this total. If they endorsed either “skill”, or “a combination of skill and chance” a one was added to this total.

*Game Experience Questionnaire* For purposes external to the goals of the current study, players reactions to the games they had played were measured using the in-game version of the gaming experience questionnaire (GEQ; IJsselsteijn et al. 2008).

*Depression Anxiety Stress Scales* The short version of the Depression, Anxiety and Stress Scales (DASS-21; Henry and Crawford 2005) was also assessed for purposes peripheral to the current investigation.

### Design

Participants played two different slot machine games on the simulator. Both games consisted of 250 spins. Players bet one credit on each of nine lines. In one game the stop button was enabled. In the other game the stop button was disabled. The order of game-type presentation was counterbalanced.

### Procedure

Once participants were confirmed to have met the eligibility criteria and provided consent, they were seated at a station with 2 adjacent laptops (one for the simulator and the other for answering survey questions). The models of both laptops used were Lenovo G530-444625U. Participants first completed the demographic items, and the PGSI. Afterward participants were introduced to the slot machine simulator. Participants were shown the pay-table and told that they would be betting one credit on each of nine lines. They were told that they could win by lining up identical symbols on any one of the nine played lines, so long as the matching symbols were placed from left to right. They were told that there were two exceptions to this left-to-right rule: (1) the violin symbol which was a scatter symbol, and (2) the stereo triplet that occurred on reels 3, 4 and 5. They were told that 1000 credits ($10.00) had been preloaded into the machine and that they could keep whatever was left at the end of 500 spins (all players finished with a balance of $7.32 CDN which was rounded up to $10.00). Participants who began with the stop button were shown how to use the stop button and were instructed to use it on every spin in that session. Skin conductance electrodes were attached at this point, and participants were instructed to keep their left hand still to the best of their ability while playing the slot machine. Following this instruction period, participants played the first block of 250 spins (with or without the stop button, counterbalanced). A pop-up message occurred at the end of 250 spins, whereupon participants were instructed that they would now complete a short questionnaire (i.e. the in-game GEQ) before playing the second version of the game (e.g., a version with or without the stop button depending on counterbalancing). The in-game GEQ was completed once more upon completion of the second block of 250 spins.

Participants were detached from the skin conductance apparatus and then completed the post-game questionnaire battery including the GCRS and erroneous cognitions items specific to the near-miss and the stop button. For purposes unrelated to the current study, participants also completed the DASS-21. Participants were debriefed, given their winnings and a $25 Walmart gift card for participating.

## Results

Of the 132 participants who had completed the PGSI, there were 26 non-problem gamblers, 63 low-risk gamblers (score of 1–4 on the PGSI), 19 moderate-risk gamblers and 24 Problem Gamblers (scoring 8 or greater on the PGSI). Seven participants withdrew from the study prior to completing all of the subjective measures leaving a sample of 125 participants for the subjective measures.

### Erroneous Cognitions Specific to Slot Machine Features

*Near-miss Erroneous Cognitions* Frequencies pertaining to how players responded to “Near-misses reflect my skill at this slots game, and that I was close to winning” are shown in Table 1 and “Near-misses indicate that a win is imminent” are also shown in Table 1. Based on these frequencies, a small but meaningful percentage of players endorse these beliefs (16% endorsed the belief that near-misses reflected skill at slots, and just over 11% believed that near-misses were a harbinger of upcoming wins). These beliefs appeared to coincide with one another—scores on the near-miss as skill item were correlated with scores related to near-misses predicting wins (*r* (123) = .792, *p* < .001).

**Table 1:** (a) Frequencies of different responses to “Near-misses reflect my skill at this slots game and indicate that I was close to winning.” (b) Frequencies of different responses to “Near-misses indicate that a win is imminent”

Response *(N* = *125)*	a		b	
	Frequency	Percent	Frequency	Percent
Strongly disagree (0)	55	44	53	42.4
Disagree (1)	36	28.8	37	29.6
Neither agree nor disagree (2)	14	11.2	21	16.8
Agree (3)	18	14.4	13	10.4
Strongly agree (4)	2	1.6	1	.80

Crucially, the near-miss as a skill item was correlated with PGSI scores (*r* (123) = .193, *p* = .031), as was the near-miss win imminence item (*r* (123) = .209, *p* = .02). Each item was also significantly related to the subscales of the GRCS as shown in Table 2.

**Table 2:** Correlations between the near-miss as skill item, near-miss as a harbinger of wins item, stop button as a harbinger of wins item, and stop button composite score with subscales on the Gambling Related Cognitions Scale (GRCS)

Item	Illusion of control	Predictive control	Interpretive bias	Stop gambling	Gambling expectancies
Near-miss as skill	*r* = .471 *p* < .001	*r* = .473 *p* < .001	*r* = .391 *p* < .001	*r* = .294 *p* = .001	*r* = .307 *p* < .001
Near-miss predicts wins	*r* = .538 *p* < .001	*r* = .546 *p* < .001	*r* = .454 *p* < .001	*r* = .298 *p* < .001	*r* = .320 *p* < .001
Stop buttons made wins more likely	*r* = .368 *p* < .001	*r* = .365 *p* < .001	*r* = .302 *p* = .001	*r* = .109 n.s.	*r* = .158 n.s.
Stop button composite score	*r* = .357 *p* < .001	*r* = .354 *p* < .001	*r* = .290 *p* = .001	*r* = .206 *p* = .021	*r* = .120 n.s.

*N. B.* The degrees of freedom was df = 123 for all correlations

*Stop Button Erroneous Cognitions* To assess the prevalence of erroneous beliefs about the stop button feature being a skill-device, we tabulated how often players endorsed the statement “Using the stop button made wins more likely.” Out of the 125 responses 43 (34.4%) strongly disagreed with the statement, 46 (36.8%) disagreed with this statement, and 19 (15.2%) neither agreed or disagreed. By contrast, a concerning total of 15 participants (12%) agreed with this statement, and 2 (1.6%) participants strongly agreed with this statement.

We also calculated a composite score based on the items from Ladouceur and Sévigny (2005), designed to assess whether participants held erroneous beliefs about the stop button. This composite score was correlated with the degree to which they endorsed the statement “Using the stop button made wins more likely” (*r* = .667, *p* < .001).

This composite score was also significantly correlated with PGSI scores (*r* = .240, *p* = .007), indicating a relationship between problem gambling status and erroneous beliefs pertaining to the stop button. Composite scores were also significantly correlated with the illusion of control, predictive control and interpretive bias scales of the GRCS (See Table 2). Further, both the single item and the composite items were unrelated to gambling expectancies, and only weakly related to cognitions about the inability to stop gambling.

Thirty-four of the 125 respondents preferred the game with the stop button feature. Independent t-tests revealed that those who preferred the stop button more strongly endorsed the idea that the stop button made wins more likely (*M* = 1.71) than those who preferred the no stop button game (*M* = .87), *t*(123) = 4.194, *p* < .001. As the question format was dichotomous, the remaining 91 respondents preferred the game without the stop button.

### In-Game Physiological Measures: Data Reduction and Analysis Strategy

Of the 132 participants, seven dropped out prior to completing both slot machine tasks. One participant’s force data and one participant’s SCR data was not recorded leaving samples of 124 for force, 124 for SCRs and 125 for PRPs. In analyzing our physiological measures, we partitioned our analysis in two ways. Firstly, we performed separate repeated measures analyses of variance (ANOVAs) for skin conductance (SCRs), force, and post-reinforcement pauses. The repeated factors were outcome type (losses, gramophone near-misses, LDWs, small regular wins, gramophone wins and stereo triplet wins and the stereo triplet near-misses) and button use (stop button, no stop button). We then conducted a more restricted version of the analyses above in which the outcomes measured were limited to the three types of losing outcomes that result in no credit gains. Post-hoc analyses were conducted using Fisher’s LSD procedure with alpha set at .01. Violations of sphericity were tested using Mauchly’s test, and in the event of significant violations we applied Greenhouse-Geisser corrections to the degrees of freedom which are reported in the analyses below. We made an a priori decision not to look at gambling status effects because no effects of gambling status had been shown to influence these in-game measures in our previous studies. For all repeated measures analyses effect sizes were measured using partial eta squared (reported as *η* ^*2*^).

**Fig. 4:**
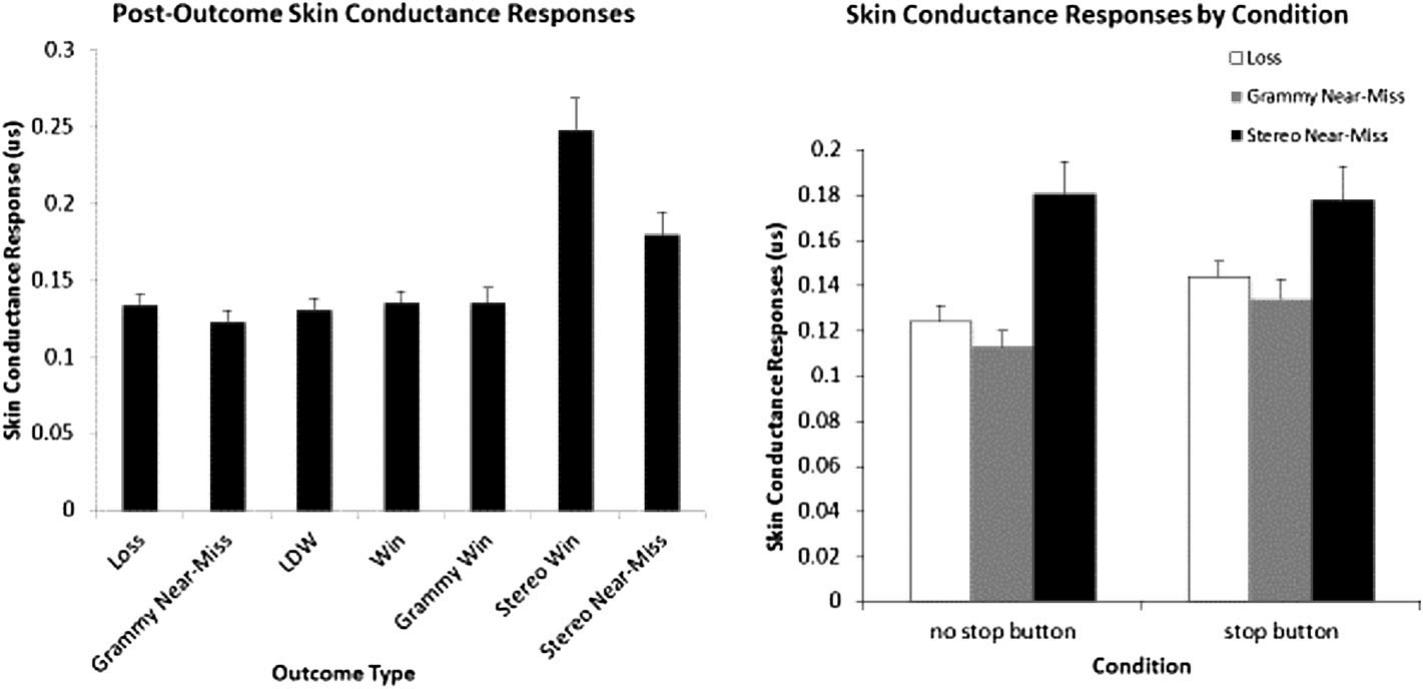
*Left panel* Average skin conductance response magnitudes following winning and losing outcomes. *Right panel* Average skin conductance responses for the full loss outcomes (credit gains of zero) in the no stop button and stop button conditions. *Error bars* ±1 SE

*Skin Conductance Responses* A repeated measures ANOVA revealed a significant main effect of button use, *F*(1, 123) = 6.550, *p* = .012, (*η* ^*2*^ = .051). Overall, SCR magnitudes were on average greater for participants when they used the stop button (*M* = .163) compared to when they played without the stop button (*M* = .147). A significant effect of outcome was also observed, *F*(1.8, 221.424) = 37.863, *p* < .001 (*η* ^*2*^ = .235), but there was no stop button use by outcome interaction (*F* (2.48, 305.394) = 1.006, *p* = .380). As shown in Fig. 4, the main effect of outcome was caused by the greater magnitude of SCRs following stereo triplet wins, and following stereo triplet near-misses. Fisher’s LSD post hoc analyses indicated that, stereo triplet wins triggered greater SCRs than all the other outcomes (all *p* values <.001). Stereo triplet near-misses triggered larger SCRs than regular losses, gramophone near-misses, LDWs, small wins and gramophone wins (all *p* values <.001). The lack of interaction indicates that using the button did not preferentially inflate the SCRs on any particular outcome.

As can be seen in the left panel of Fig. 4, the five outcomes on the left side of the graph (losses, gramophone near-misses, LDWs, small wins and gramophone wins) triggered small SCRs that were relatively similar in magnitude. Among these outcomes the only statistical difference was that (inexplicably) gramophone near-misses had significantly smaller SCRs than losses—none of the other contrasts were significant.

When analyzing SCRs for outcomes that resulted in no credit gain (losses, gramophone near-misses, stereo triplet near-misses), there was a main effect of button use, *F*(1, 123) = 4.951, *p* = .028, (*η* ^*2*^ = .039) and a main effect of outcome, *F*(1.143, 140.627) = 28.695, *p* ≤ .001 (*η* ^*2*^ = .189). There was also a significant outcome by button use interaction, *F*(1.752, 215.528) = 4.699, *p* = .013 (*η* ^*2*^ = .037). Average SCRs following losing outcomes are displayed in the right panel of Fig. 4. The main effect of outcome is attributable to the greater magnitude of SCRs for stereo triplet near-misses, compared to gramophone near-misses or regular losses. For the no stop button condition, there was a large main effect of outcome *F*(1.223, 152.884) = 29.837, *p* < .001, (*η* ^*2*^ = .193) with stereo near-misses having more robust SCRs than losses (*p* < .001) and gramophone near-misses (*p* < .001). Similarly, in the stop button condition the effect of outcome was also highly significant, *F*(1.345, 166.797) = 14.738, *p* < .001 (*η* ^*2*^ = .106) with stereo triplet near-misses significantly larger than either gramophone near-misses (*p* < .001) or losses (*p* < .001) in the stop button condition. As can be seen in the right panel of Fig. 4 (and the effect sizes for the simple main effects reported above), the interaction was caused by the larger differences between the stereo triplet near-misses and regular losses and gramophone near-misses in the no stop button condition.

**Fig. 5:**
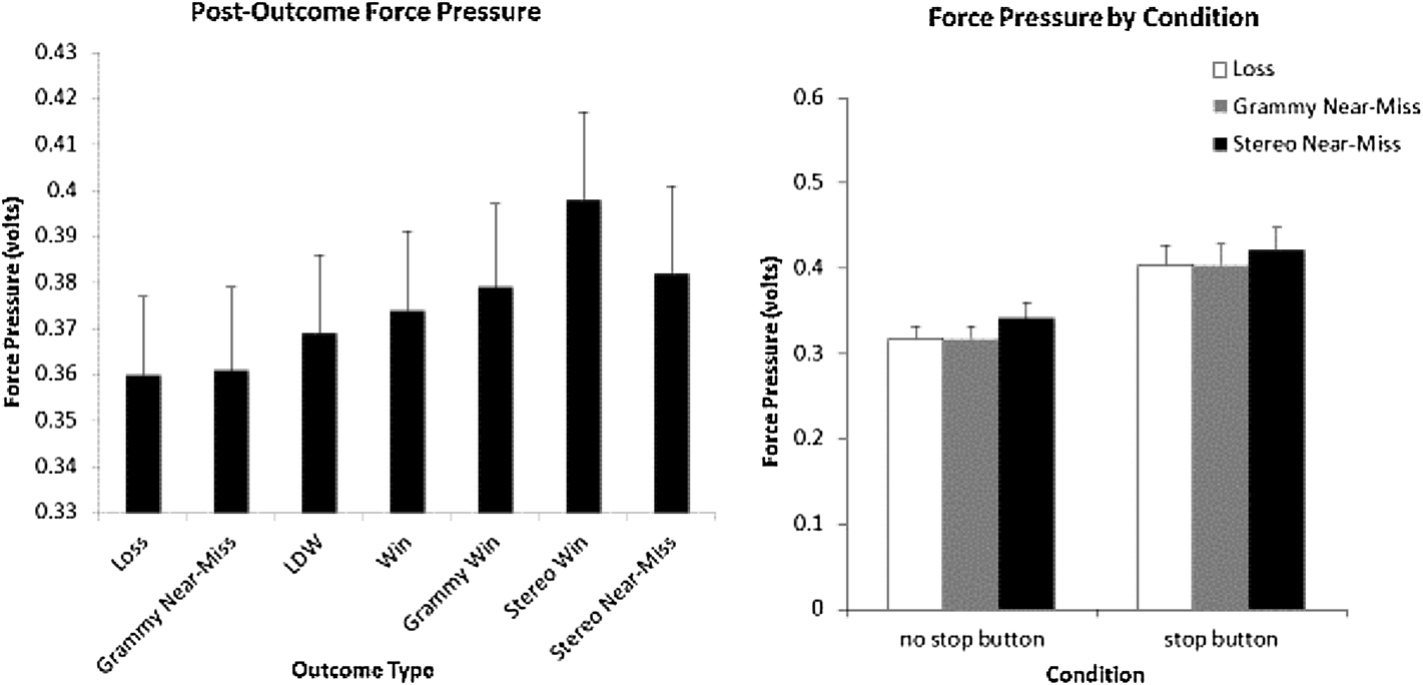
*Left panel* Average force (v) applied to the spin-button following losing and winning outcomes. *Right panel* Force pressures (v) used to initiate next spins for full loss outcomes (credit gains of zero) in the no stop button and stop button conditions. *Error bars* ±1 SE

*Force Applied to the Spin-Button* A repeated measures ANOVA revealed a significant main effect of button use *F*(1, 123) = 21.39, *p* < .001, (*η* ^*2*^ = .148). Greater force was applied to the spin-button in the stop button condition (*M* = .416) compared to the no stop button condition (*M* = .333). A main effect of outcome was also observed, *F*(3.68, 453.040) = 16.468, *p* < .001 (*η* ^*2*^ = .118), however, there was no button by outcome interaction, F(3.217, 402.323) = 1.494, *p* = .212). Fisher’s LSD Post-hoc comparisons indicated that players elicited greater magnitudes of force following LDWs compared to regular losses (*p* = .001) as well as gramophone near-misses (*p* = .004). Stereo wins triggered more force than any other outcome (largest *p* = .008). Crucially, as shown in the left panel of Fig. 5, stereo triplet near-misses triggered more force than LDWs (*p* = .002), gramophone near-misses (*p* < .001) and regular losses (*p* < .001), and equivalent amounts of force as small wins, and gramophone wins.

When restricting the analysis to just the losing outcomes (losses, gramophone near-misses and stereo triplet near-misses), a main effect of button *F*(1, 123) = 20.388, *p* < .001 (*η* ^*2*^ = .142), and a main effect of outcome, *F*(1.423, 175.029) = 22.532, *p* < .001, (*η* ^*2*^ = .155) were revealed. However, there was no button by outcome interaction. The main effect of button use was attributed to players applying more force to initiate the spin button in the stop button condition (*M* = .410) compared to the no stop button condition (*M* = .325). As illustrated in the right panel of Fig. 5, the main effect of outcome was attributable to players initiating the next spin with greater force after stereo triplet near-misses than after gramophone near-misses (*p* < .001), or after regular losses (*p* < .001). Gramophone near-misses were not significantly different from regular losses (*p* = .942).

**Fig. 6:**
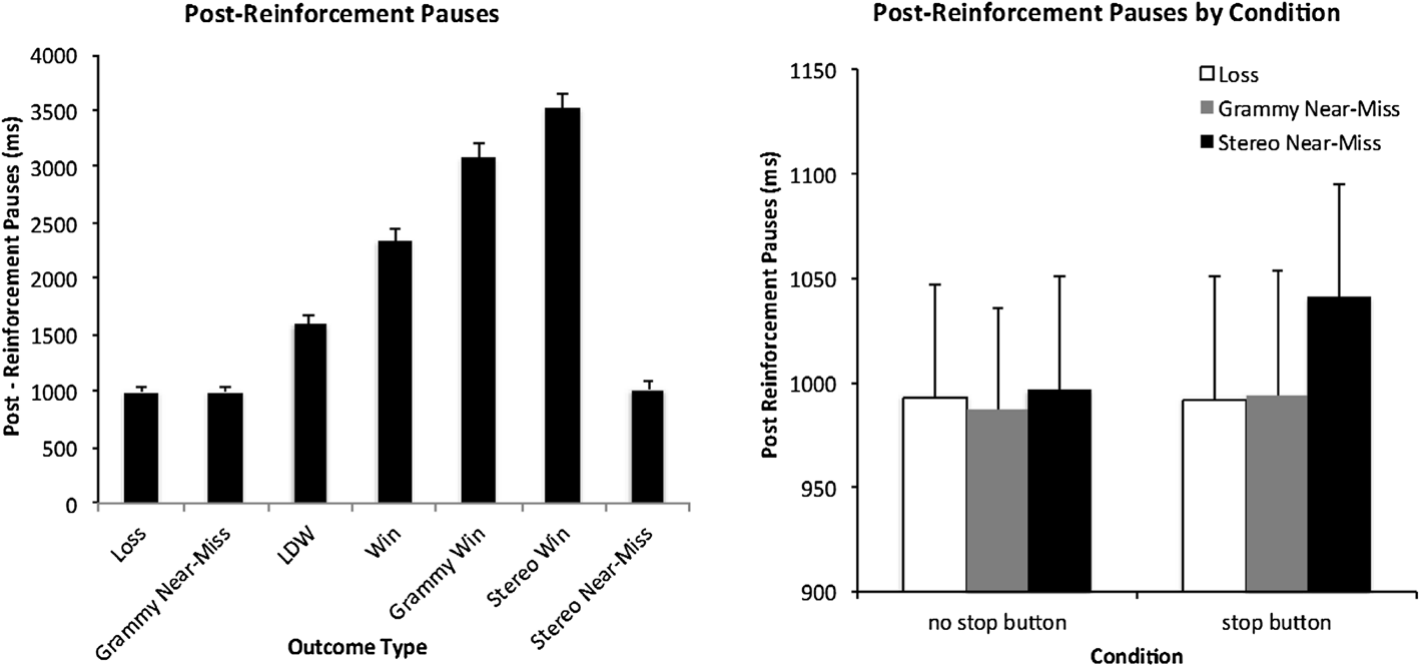
*Left panel* Average post-reinforcement pauses (ms) following winning and losing outcomes. *Right panel* Average post-reinforcement pauses (ms) for full loss outcomes (credit gains of zero) in the no stop button and stop button conditions. *Error bars* ±1 SE

*Post-reinforcement Pauses* The repeated measure ANOVA for PRPs observed no significant main effects of stop button use *F*(1, 124) = .937, *p* = .335. However, there was a significant main effect of outcome, *F*(1.286, 159.458) = 455.039, *p* < .001 (*η* ^*2*^ = .786), while the button by outcome interaction fell just short of significance following a Greenhouse Geisser correction *F*(2.058, 255.186) = 2.82, *p* = .06. The main effect of outcome is shown in the left panel of Fig. 6. Post-hoc analyses showed that all outcomes were different from one another (all p values < .001) except the losses, the gramophone near-misses and the stereo triplet near misses.

The restricted analysis of PRPs for the three types of full losses (losses, gramophone near-misses, stereo near-misses), showed that the main effects of both button use and outcome were not significant. Crucially however, there was a significant button use by outcome interaction, *F*(1.724, 213.746) = 5.118, *p* = .01, (*η* ^*2*^ = .040). Simple effect analyses revealed that there was a main effect of outcome only in the stop button condition, *F*(1.379, 172.385) = 6.245, *p* = .007, (*η* ^*2*^ = .048) and not the no stop button condition. As shown in the right panel of Fig. 6, this interaction appears to be attributed to higher PRPs following stereo near-misses, but only when players were using the stop button. Fisher’s LSD post hoc comparisons revealed that while losses and gramophone near-misses did not statistically differ (*p* = .820), the stereo near-misses had longer PRPs than both losses (*p* = .008) or gramophone near-misses (*p* = .011) in this stop button condition.

## Discussion

In the present study, players played two separate games on a slot machine simulator: one involving the use of the stop button, and the other without the use of a stop button. We sought to assess whether two distinct structural slot machine features, the stop button and near-miss outcomes, would perpetuate a unique set of erroneous cognitions and amplified emotional responses related to the presence of such erroneous cognitions.

Perhaps not surprisingly, the use of the stop button was affiliated with erroneous cognitions related to skill and the ability to influence the outcomes of the game. Indeed, erroneous cognitions related to the stop button were specifically correlated with the illusion of control and predictive control subcomponents of the Gambling Related Cognitions Scale. When asked about the role of the stop button during play, 13.6% of the sample agreed that the stop button was related to the probability of winning. This is a troubling percentage of players when one considers that signage in Ontario casinos explicitly indicates that the stop button does *not* influence the outcomes on the machines. Critically, our composite “stop button skill” score (based on the items used by Ladouceur and Sévigny 2005) was significantly correlated with problem gambling severity. This relationship supports previous research showing that skill-oriented beliefs in games of chance are core cognitive processes that shape problem gambling behaviour (Griffiths 1994; Myrseth et al. 2010).

The use of the stop button impacted how players actually played these games. It impacted how players reacted to both winning and losing outcomes of the games. Our analysis of the force which players initiated spins indicates that for all outcomes players used more force to initiate the next spin when using the stop button. They also showed larger SCRs for all outcomes (except for the stereo near-misses) when using the stop button. It would seem that they literally played the game “harder”. One interpretation of this pattern of results is that that players may be exerting a strategy (albeit an ineffective one) to stop the reels in a winning combination, presumably by “precision” timing and effort. If players were merely using the stop button to reveal the outcome faster, there is no reason to assume they would pull the stop button especially hard. If, however, players were attempting to stop the reels at a given outcome, then they would likely pull hard to get the reels to stop spinning at a winning combination. The arousal and effort used to stop reels at a winning combination could then bleed over into the initiation of the next spin, causing players to initiate spins with greater force in the stop button condition than the no-stop button condition. Thus the finding of greater SCRs and greater force in the stop button condition may constitute in-game evidence of the erroneous cognitions we saw using the post-game questionnaires where at least some gamblers thought that using the stop button was related to skill at slots. Although in our simulator (and in actual slots games), stop buttons merely reveal the outcome faster, our force results suggest that players feel they can impact the outcomes of the games by pulling at just the right time. As such our force and SCR results may actually reflect the illusion of control during play.

Another notable aspect of our force measures is that unlike SCRs, force pulls appeared to be quite sensitive to the amount of credits gained. Whereas the losses, LDWs and small wins, and even the large gramophone win all led to roughly equivalent SCR responses (see the first five outcomes listed in the left panel of Fig. 4), our force measure was able to more effectively differentiate these outcomes (note the systematic increase in force with win size in the left panel of Fig. 5). Thus force appears to be a more sensitive measure than SCRs to win size—at least for this game, and for an interface that involves players pulling on a spin/stop button as opposed to pushing a spin/stop button. This finding may be quite important when one considers ecological validity. With SCRs, participants have to be repeatedly told not to move their non-dominant hand lest they mar the SCR recordings. The visual presence of the electrodes on their fingers, the wires connecting the electrodes, along with such movement constraints, may prevent players from becoming as absorbed as they normally would in the slots games. By contrast the spin/stop button is simply part of how they play the game and is at least in this experiment, more sensitive than SCRs at measuring players reactions to outcomes that differ in terms of the credits gained. As such it may represent a very effective tool in the gambling researchers’ armamentarium. Of course, further validation is needed to confirm the sensitivity of the force measure to appetitive and frustrative reactions.

All three in-game measures converged to suggest that players erroneously interpret near-miss outcomes as conveying something very different than regular losses. When stereo triplet near-misses were compared to losses, they triggered significantly greater SCRs in both the stop button and no-stop button condition suggesting that players reacted to these outcomes with more frustration than regular losses. This interpretation was bolstered by our measures of force to initiate the next spin. In both the stop button and no-stop button condition, players used greater force to initiate the next spin following near-misses than following regular losses.

Another impactful finding concerned players’ PRPs following the stereo near-misses. In the no-stop button condition PRPs for the highly salient stereo near-misses were equivalent to other losing outcomes and far shorter than any winning outcome. This finding is consistent with our previous work (Dixon et al. 2013, 2015) but runs counter to studies by Dixon and Schreiber (2004) and Belisle and Dixon (2016) which showed that PRPs following near-misses were more similar to wins than standard losses. In contrast to play without the stop button, significant PRP differences between stereo near-misses and regular losses did occur when players used the stop button. It is important to note that in both button conditions the PRP was measured in the same way (from the time of the last reel stopping and the initiation of the next spin). Although one might suspect that an action immediately prior to this measurement (i.e., pressing the stop button) might perturb ensuing PRPs, we note that the PRP was only affected following salient near-misses. As shown in Fig. 6 the PRPs for regular losses were virtually identical in both the button use and no button use condition. As such our findings are unlikely due to the stop button interfering with PRPs in a general sense. Although the near-misses in the stop button were significantly longer than other types of full losses, they were still more similar to these losses than to wins (i.e., near-misses in the stop button condition were 49 ms longer than full losses, but were over 500 ms shorter than LDWs and over 1000 ms shorter than small wins). Thus, the explanation that players are misinterpreting near-misses as wins does not seem to translate to our findings. Rather, it may be that the small increase in length of the PRP for stereo near-misses when players use the stop button is attributable to a brief skill-related evaluation period. This may also account for the pattern of SCRs shown in Fig. 4. While SCRs were higher in the stop button compared to the no stop button condition for losses, gramophone near-misses, LDWs, small wins, gramophone wins and stereo triplet wins, they were equivalent for stereo triplet near-misses. It may be that skill-related mentations may have curtailed the arousal due to frustration in this key condition.

Overall our interpretation of the SCR, PRP and force findings is that at least for some players the stop button is encouraging a perception of skill not otherwise present in the games without the stop button. As such, it is possible that players are more attuned to perceiving the near-misses as a form of performance feedback. As players invest a great deal of “effort”, evidenced by the mere action of pressing a stop button while reels are spinning, they may experience a near-miss outcome as indicative of their improved performance of “getting closer to their goal”. If they perceive themselves to be personally responsible for this near-miss, one might expect a longer pause to evaluate their proximity to this desired win. Longer PRPs associated with near-misses in the stop button condition may reflect an increase in the “wanting” component of the reward system as opposed to a “liking” component of the reward system (Berridge 2004). The latter liking component would of course be indexed to the very long PRPs, high SCRs and large force associated with the exciting stereo triplet wins.

Consistent with Dixon et al. (2015), when comparing player responses to the different outcomes it is clear that even in complex multiline games, certain symbols (the horizontal triplets) can be made to stand out to the player. For all measures in the current study, the horizontal triplet wins led to an enhanced response over and above the gramophone win, even though both win types were equated in terms of value. The finding that players showed significantly larger SCRs, force and PRPs following the stereo horizontal triplets compared to the gramophone wins shows that slot machine game designers can sensitize players to certain symbols over others. More importantly, once players become sensitized to these symbols, game designers can then elicit near-miss effects when players “just-miss” a winning combination with these highly salient triplet symbols. While near-miss effects have often been shown in single-line games, this study replicates the findings of Dixon et al. (2015), and conclusively shows that near-miss effects can be demonstrated in multiline games—a finding that is important given the burgeoning popularity of these games in modern casinos.

### Limitations

Although our data pertaining to erroneous cognitions are telling in terms of the nature of multiline slots play, there are still a few lingering questions that could not be answered due to limitations necessitated by the study’s design. The slot machine simulator was played on an ordinary laptop computer, which lacks many of the cues and capabilities of an actual slot machine. Furthermore, having players interact with the machine using a modified mouse which they pulled, may elicit different effects (especially on force measures) than a slot machine simulator game housed in a realistic cabinet where players push on a spin/stop button located on the front panel. Additionally, our measures involving skin conductance where participants had electrodes attached to their fingers of their non-dominant hand, and were instructed not to move said hand, could affect how absorbed players become in slots play. Such playing conditions are novel and unnatural for any slot machine player. Also the rather standard limitation for studies of this type, namely that players were not using their own money certainly applies here. Despite this, we were still able to detect robust changes in participants’ physiological and behavioural reaction patterns to different outcomes and show clear effects of both stop button use, and reactions to near-misses. It may be that the robust effects shown would be even more prominent in more naturalistic settings.

## Conclusion

The current study makes two novel contributions: First we move beyond the classic near-miss effects that have been demonstrated in simplistic, three-reel slot machines. Here we show that near-misses can dramatically affect player frustration and arousal even despite the complexity of the winning (and losing) symbol alignments in multi-line games. This extension is important given that multiline games appear to be the games of choice for seasoned gamblers (Livingstone et al. 2008). Second, taken together, our behavioural (i.e., force, PRPs) and physiological (i.e., SCRs) measures converge to demonstrate that using the stop button creates an illusory perception of skill that impacts how players respond to the outcomes they receive while playing the slot machine. In general, players elicit greater levels of arousal for a vast majority of the outcomes, and a more amplified frustration response to winning and losing outcomes. Our findings complement earlier work by Ladouceur and Sévigny (2005), and suggest that the use of the stop button facilitates erroneous cognitions that are detectable both behaviourally and physiologically.
